# Relationship satisfaction and self-esteem in patients with breast cancer and healthy women: the role of expected and actual personal projects support from the partner

**DOI:** 10.1186/s12905-023-02555-1

**Published:** 2023-08-11

**Authors:** Sára Imola Csuka, Judit Désfalvi, Barna Konkolÿ Thege, Viola Sallay, Tamás Martos

**Affiliations:** 1 Institute of Psychology, University of Szeged, Szeged, Hungary; 2 Schools of PhD Studies, Semmelweis University, Budapest, Hungary; 3 1st Department of Internal Medicine and Oncology, Semmelweis University, Budapest, Hungary; 4 Waypoint Research Institute, Waypoint Centre for Mental Health Care, Penetanguishene, ON Canada; 5 Department of Psychiatry, University of Toronto, Toronto, ON Canada

**Keywords:** Breast cancer, Self-Determination Theory, Personal projects support, Relationship satisfaction, Self-esteem

## Abstract

**Background:**

For breast cancer patients, the partner’s support for personal projects can serve as a means of adaptation. We aimed to investigate the associations between the intimate partner’s personal project support and women’s well-being.

**Methods:**

A sample of 274 Hungarian women (breast cancer patients *n* = 137, control *n* = 137) took part in the study. Expected and actually received autonomy-, directive- and emotional project support was assessed by the procedure of Personal Project Assessment. Well-being was measured by the Relationship Assessment Scale and the Rosenberg Self-Esteem Scale. For investigating the associations between project support and well-being in a multivariate way, structural equation modelling was used.

**Results:**

Except for autonomy support, participants expected more support than they received. A path model indicated multiple associations between types of project support and relationship satisfaction and self-esteem. The partner’s emotional project support was predictive of women’s relationship satisfaction and self-esteem, while directive support was predictive of self-esteem only. The associations showed similar patterns in the subgroups of patients with breast cancer and control.

**Conclusions:**

Our results highlight the importance of involving women’s subjective perspectives regarding the partner’s project support while also have implications for praxis. Teaching women how to communicate their needs to their partner effectively (whether it is the need for autonomy or directive guidance) can help close the gap between expected and received support, which may in turn enhance relationship satisfaction and self-esteem.

## Background

### Introduction

Breast cancer (BC) is the most frequent carcinoma disease, with a worldwide prevalence rate estimated by WHO to be 2.26 million cases in 2020. The present study was conducted in Hungary, where the prevalence rate of BC is relatively high among the European countries, with a proportion of 131 cases per 100 000 in 2020 [1]. Accordingly, BC is a considerable public health concern in Hungary [2]. Challenges for women also include facing the insecurity of the future, as well as maintaining autonomy and healthy relationships [3]. Hence, the intimate partner’s support is essential for adaptation to the illness. In a crisis, most people seek help from their closest relationships because these ties give emotional support and strengthen self-esteem [3]. Among supportive relationships, an intimate relationship has unique significance for women because of its relative stability and emotional intensity [4], and during an illness, it may gain even higher significance [5]. Moreover, the diagnosis of cancer can affect everyday preferences and induce shifts in personal goals (i.e., personal projects [6]).

Therefore, our aim was to investigate the relationship between different forms of the partner’s goal support on the one hand, and relationship satisfaction and self-esteem on the other hand among breast cancer patients and a control group of women without a cancer diagnosis, using an observational and comparative study design. The inclusion of a control group was essential to explore whether specific psychosocial characteristics associated with cancer (e.g. increased importance of partner support, possible changes in communication styles) cause differences compared to women without BC. Moreover, there is a lack of studies using a control group for comparing patients with BC to a healthy control group [7–9], and we are not aware of any studies comparing partner’s goal-related support among women with and without BC.

### Self-determination theory’s account on adaptation to a chronic illness

Self-Determination Theory (SDT) is a comprehensive motivational theory focusing on well-being and emphasizing the dynamic interrelation of the person’s inherent growth potential and the circumstances given by the social environment [10]. By considering these components together, the theory can be applied to the examination of close relationships [11] thus, SDT has also been widely applied to study chronic illness adaptation [12], including cancer (e.g. Brunet et al., 2013 [13]; Milne et al., 2008 [14]). SDT postulates three basic psychological needs: the need for autonomy (i.e., striving for personal volition and self-sufficient decisions), the need for competence (i.e., having knowledge and skills necessary for an activity), and the need for relatedness (i.e., the need for being connected to others). According to SDT, the satisfaction of these needs leads to well-being and internalization of a given behaviour, in contrast to when the environment does not allow for the satisfaction of these needs, it may lead to negative consequences in terms of health and integrity [10]. The support for autonomy and emotional relatedness seems especially important from the partner of women struggling with BC.

Autonomy support is connected to offering the possibility of choice in illness management [15]. Thus, the partner’s support can also increase self-esteem [3]. Self-esteem has been shown to be a key psychological resource in cancer as it is associated with better adjustment to the illness and it is a protective factor against depression; however, the physical damages caused by treatments of cancer and fertility-related concerns in women can impair self-esteem [16, 17]. Emotional support is another key characteristic of the supporting partnership as it promotes the elaboration of feelings accompanying the illness, while the recognition of feelings enables the internalization of health-related goals and actions. According to previous studies involving patients with BC, there is a tendency to have a higher need for expressing self-conscious emotions (compared to their partners who often try to protect them and hold back emotions) [18]. In a study of women with BC, participants were more satisfied with their relationship when their partners validated the expression of their feelings and acknowledged their point of view [19].

### Personal projects and chronic illness

Personal projects are specific mid-term goals and plans that a person is actively pursuing in the present [20], for example, take a language course, strengthen family relationships or buy a coat for their child. Personal projects are interrelated with actual health status and behavior (e.g. [21, 22]), and, therefore, striving for personal projects can facilitate illness adaptation and well-being (e.g. [23]). Conversely, a life-threatening chronic disease like cancer inevitably affects the patient's personal projects [6]. Moreover, since personal projects are embedded in a social context [20], the partner’s support for everyday projects can enhance physical and psychological well-being [24]. However, with a few notable exceptions, support of personal projects during illness is rarely studied. In a study with adolescents with cancer, the diagnosed group had more social support than the control group, especially for goals they established in response to cancer [23]. In a study with stroke patients, those who received more intensive peer support in their strivings showed higher levels of well-being 24 months after the diagnosis [25]. Chow [26] came to a similar conclusion concerning patients with BC: the partner’s emotional support was identified as a factor that facilitated project pursuit.

According to SDT, different forms of support are not equally associated with well-being. For women during the physical and mental challenges of BC, the partner can be the source of support but may also undermine self-determination [27, 28]. Directive goal support can be defined as giving reminders, advice and direct suggestions on how the person should act in a given situation [29, 30]. Moreover, directive goal support is unrelated or negatively associated with well-being [30, 31]. A study found that trying to persuade the partner and using reminders or compliments for behavior change facilitated health behavior in couples [32].

In addition, well-being may be determined by personal expectations of support (based on the basic psychological needs) above the support actually given [27]. However, in a relationship, the need to discuss difficulties and express feelings as well as strive to protect the partner (e.g. hiding concerns to spare the partner emotionally) is not always in equilibrium [18]. Despite this assumption, we are not aware of studies examining the amount of expected and received goal support from the partner simultaneously. Hence, such investigations would be of utmost importance as, on the one hand, those being in a less favorable physical condition perceive lower levels of social support [33]; moreover, they are exposed to the partner’s avoidance or social restrictions to a greater extent [34]. On the other hand, when the physical status of women with BC becomes worse, compassionate communication from the partner gains special significance in reducing distress [19]. Consequently, in the case of BC, there can be an increased need for support, while women’s subjective perspectives might largely influence the perception of the partner’s actual responses.

### The present study

In the present study, we apply a self-determination theory approach [10] to investigate women’s personal project pursuit, expected and received support for their projects, in relation to their relationship satisfaction and self-esteem. First, we address the potential differences between women with BC diagnosis and a control group. We have developed two testable research questions without forming explicit hypotheses about the strength and direction of the associations.

- Q1. We tested if there was a difference a) between the level of support expected and received from the partner while accomplishing personal projects and b) in what ways these types of support predict relationship satisfaction and self-esteem of women.
- Q2. We examined if there were differences between the two study groups regarding a) received and expected project support, b) levels of relationship satisfaction and self-esteem, and c) associations between support and relationship satisfaction and self-esteem.

Second, we investigated the associations between different types of partner support for personal projects (i.e., autonomy, directive, and emotional support) and women’s relationship satisfaction and self-esteem. Third, we studied the differences between the amount of the expected and actually received support from the partner (received support based on the subjective perception of women).

Concerning the second and third aims of the study, we tested the following hypothesis.

- H1. Higher perceived autonomy and emotional support from the partner in the personal projects are positively associated, while directive support is negatively associated with relationship satisfaction and self-esteem.

## Methods

### Participants and procedure

The study aimed to examine samples of Hungarian women with and without BC. For this aim, we recruited a total sample of 274 Hungarian women, of whom 137 were diagnosed with BC within the last year. A sample size of *N* = 123 was calculated with an alpha level of 0.05 and a statistical power of 0.80 to detect a correlation coefficient of 0.25; therefore, we considered the obtained sample size appropriate for the study. Non-diagnosed healthy respondents were selected to fit the diagnosed group by age to serve as a matched control group (*n* = 137). The inclusion criteria for both groups were to live in a romantic relationship (in the patient group, at least three months prior to receiving the diagnosis). Exclusion criteria were having any other chronic illness or being under psychiatric treatment.

Patients with BC were contacted through an Oncology Centre in Budapest, while members of the control group were selected from a larger database of non-diagnosed, community-dwelling women being part of a study on support for personal projects and well-being. The study was approved by the Regional Institutional Scientific and Research Ethics Committee at Semmelweis University (# 98–2/2014). Participation in the study was voluntary and anonymous, and respondents signed a written informed consent after they were provided with sufficient information about the main aims of the study and the confidential nature of data management. The two groups responded to the same test battery in a paper–pencil format, which took approximately 35 min to complete.

### Measures

The survey started with general questions (demographics, education level, self-rated health status, and relationship status) and consisted of two major units. The first part focused on personal project assessment and project support from the partner. The second part contained the measures of relationship satisfaction and self-esteem.

### Assessment of personal projects

The goals of the participants were assessed using a the procedure that was informed by the Personal Project Assessment (PPA) method [20]. Following the standard PPA procedure, participants were asked to identify and list all their personally relevant projects in the first step. In the next step, they were instructed to choose their four personally most important projects from the list regarded as the most important personally. Finally, each of these four chosen projects was rated on a 7-point Likert-type scale along the same aspects concerning the nature (autonomy, directive, and emotional support) and extent of support *a) expected* and then *b) actually received* from the partner. Since previous research did not assess partner support in terms of both expected and received support and their combination with the type of support (autonomy, directive, and emotional), to address the gap, we developed a new set of personal project items specifically for this study that covers these aspects. It is important to note that personal project assessment is a flexible process that can be customized to measure various types of experiences and evaluation criteria, depending on the focus of the research being conducted [34].

To assess *expected support* for the personal projects, we used the prompt “I expect my partner … “ and provided the following complex descriptions to assess each type of support: 1) autonomy support: “… to entrust me how I get on with this project and how I would like to realize it; to let me decide freely about it.”; 2) directive support: „… to ask me often about how I get on with this project; to suggest steps and remind me what to do.”; 3) emotional support: „… to stand by me emotionally in this project, so that I can feel his acceptance and care.”

To assess *perceived* support, we used the prompt “My partner …” and provided the slightly modified versions of the descriptions above to assess the amount of autonomy support (“…entrusts me how I get on with this project and how I would like to realize it; lets me decide freely about it.”, directive support („…asks me often about how I get on with this project; suggests steps and reminds me what to do.”), and emotional support (“…stands by me emotionally in this project, so that I can feel his acceptance and care”). Ratings of the six (2 × 3) support assessments were averaged across the four projects.

#### Relationship satisfaction

Relationship satisfaction was measured by the Relationship Assessment Scale ([35]; Hungarian version: [36]). The measurement consists of 7 items, e.g., “How well does your partner meet your needs?” Women had to answer the questions using a five-point Likert-type scale. The scale had good internal consistency in the present sample (Cronbach’s α = 0.82).

#### Self-esteem

Self-esteem was captured by the Rosenberg Self-Esteem Scale ([37]; Hungarian version: RSES-H [38]), which is a self-report measure that pertains to self-acceptance and self-worth. It comprises ten statements, e.g., “On the whole, I am satisfied with myself.” Respondents were asked to rate on a four-point Likert-type scale to what extent the statement described their self-esteem. The scale had excellent internal consistency in the present data set (Cronbach’s α = 0.88).

### Statistical analyses

In the first step, differences between the two groups were examined. As the sample size was large (*n* = 137 for both groups), according to the central limit theorem [39], distribution was regarded as normally distributed; thus, parametric tests were applied. Independent-samples t-tests and Kolmogorov–Smirnov Z-test were used to examine group differences. Pearson correlation coefficients were calculated to capture bivariate relationships between the studied constructs. Differences between the correlation coefficients of expected and perceived support’s associations with relationship satisfaction and self-esteem were tested using Fisher-z transformations.

Structural equation modelling was used to investigate the associations in a multivariate way. Model fit was tested using the following indices [40]: Chi-square statistic (χ^2^), relative Chi-square (χ^2^/df), comparative fit index (CFI), normed fix index (NFI), incremental fit index (IFI), Tucker Lewis Index or Non-normed Fit Index (TLI or NNFI), and the root mean square error of approximation (RMSEA). Among the absolute fit indices, Chi-square statistics should optimally result in a non-significant probability value at a 0.05 threshold, Relative Chi-square’s recommended range should ideally be less than 2.00 but not more than 5.00, and for RMSEA value, it should be close to 0.06 or less. Comparative fit indices compare the Chi-square value to a baseline model (null hypothesis meaning that all of the studied variables are uncorrelated); accordingly, CFI, NFI, TLI (or NNFI) values higher than 0.95 can be recognized as indicative of good fit.

## Results

### Descriptive statistics and group comparisons

The mean age of the participants was 51 years (SD = 9.58, SE = 0.58; range: 30–80 years). Most respondents (66.4%) were white-collar workers (e.g., teachers, accountants, doctors, or office workers). In terms of educational attainment, 58.9% of participants had postsecondary education, 26.7% had secondary education, and 14.5% had primary education. Regarding relationship status, 68.2% of the respondents were married, 20.2% lived in a common-law relationship, and 11.6% were in a relationship without cohabitation. Women with BC had a higher level of education than the control group (Kolmogorov–Smirnov Z = 1.79, *p* = 0.003, d = 0.153). Slightly more than half of the respondents rated their health status (on a Likert scale: 1–5) good or very good (51.5%), 39.6% were moderately satisfied, and only 8.9% reported poor or very poor health status. Women having a BC diagnosis rated their health status somewhat poorer relative to the control group (Kolmogorov–Smirnov Z = 2.31, *p* < 0.001, d = 0.2).

The level of autonomy-, directive-, and emotional support were compared in the two groups (Table 1), both for expected and actually received support types. The only difference between women with BC and the control group was that the diagnosed group expected and received a higher level of autonomy-support from their partner, and these differences had moderate effect sizes (d = 0.38 for expected and d = 0.31 received support).

**Table 1:** Descriptive statistics of the studied variables and group differences

	Diagnosed		Control		Group differences (β coefficients)	Diagnosed	Control
	Mean	SD	Mean	SD		Within–person differences (expected versus perceived support)	
Autonomy support							
Expected	5.00	1.32	4.55	1.07	-3.085* (d = 0.38)	-4.714* (d = -0.40)	-6.984* (d = -0.59)
Received	5.60	1.17	5.26	1.00	-2.601 (d = 0.31)		
Directive support							
Expected	3.70	1.46	3.86	1.34	0.950 (d = 0.11)	1.268 (d = 0.14)	1.668 (d = 0.14)
Received	3.54	1.49	3.70	1.35	0.913 (d = 0.11)		
Emotional support							
Expected	5.82	1.36	5.80	1.01	-0.164 (d = 0.02)	5.037* (d = 0.43)	3.669* (d = 0.31)
Received	5.25	1.70	5.39	1.41	0.794 (d = 0.09)		
RAS—Relationship satisfaction	27.42	6.51	29.49	5.17	2.899** (d = 0.35)	-	-
Self-Esteem Scale	31.75	5.79	32.33	4.64	0.914 (d = 0.11)	-	-

For multiple testing, Bonferroni Correction of the *p* values were used: between-person differences: * *p* < 0.016; group differences: * *p* < 0.006

Within-person differences concerning expected versus received support were also investigated. According to the results of a series of paired-samples t-tests, there were significant differences between the number of various types of expected and actual support: women in both groups expected more emotional support, while they expected less autonomy support than what they received.

Women with BC showed lower levels of relationship satisfaction relative to the control group. Pearson–correlation coefficients (Table 2) show that different support types are interrelated with relationship satisfaction and self-esteem differently but these differences show similar patterns in both groups. The overall pattern of the results shows somewhat stronger associations in the case of received relative to expected support types. Among the support types, emotional and directive support shows the strongest relationships with relationship satisfaction and self-esteem, and the associations are the most expressed in the case of emotional support and relationship satisfaction. Furthermore, Fisher-z transformations were used for testing differences between the correlation coefficients of expected and received support’s associations with well-being (Table 3). Actually received support tends to have a stronger relationship with relationship satisfaction and self-esteem than expected support.

**Table 2:** Pearson–correlation coefficients among the support and well-being variables

		1	2	3	4	5	6	7	8	
Diagnosed	1 Expected autonomy support	-	0.349^***^	0.191^*^	0.038	0.056	-0.025	-0.191^*^	0.100	Control
	2 Received autonomy support	0.292^**^	-	0.080	0.133	0.170^*^	0.289^**^	0.119	0.104	
	3 Expected directive support	0.025	-0.090	-	0.650^***^	0.408^***^	0.256^**^	0.101	-0.030	
	4 Received directive support	-0.129	0.098	0.517^***^	-	0.337^**^	0.512^**^	0.255^**^	0.185^*^	
	5 Expected emotional support	0.085	0.173^*^	0.529^***^	0.369^***^	-	0.482^***^	0.119	0.197^*^	
	6 Received emotional support	-0.044	0.345^***^	0.181^*^	0.539^***^	0.638^***^	-	0.606^***^	0.332^***^	
	7 Relationship Satisfaction	-0.128	0.225^**^	0.161	0.410^***^	0.463^***^	0.777^***^	-	0.323^**^	
	8 Self-Esteem	-0.019	0.163	-0.174^*^	0.127	0.080	0.287^**^	0.319^***^	-	

^*^ *p* < 0.05; ** *p* < 0.01; *** *p* < 0.001

Below diagonal: Diagnosed; Above diagonal: Control

**Table 3:** Fisher-z transformations for comparing the strength of correlational coefficients of expected and received support and relationship satisfaction and self-esteem

Goal support	Relationship Satisfaction (r)	z-Score	Self-Esteem (r)	z-Score
Autonomy support				
Expected	-0.180^**^	3.84 (*p* < 0.001)	0.017	-1.29 (*p* = 0.196)
Received	0.148^*^		0.128^*^	
Directive support				
Expected	0.144^*^	-2.52 (*p* = 0.012)	-0.107	-3.04 (*p* = 0.002)
Received	0.347^**^		0.154^*^	
Emotional support				
Expected	0.328^**^	-4.36 (*p* < 0.001)	0.123^*^	-2.24 (*p* = 0.025)
Received	0.703^**^		0.307^**^	

^*^p < 0.05; ^**^p < 0.01

### Hypotheses testing

Path analysis was conducted to portray the complex relationships in a multivariate way. The predictors in the model were support-type variables (expected and received), while relationship satisfaction and self-esteem were the outcome variables. Since, according to the preliminary analyses, the two study groups differed significantly in some aspects, an additional multigroup analysis was run to examine differences concerning the relationship between project support and relationship satisfaction and self-esteem. As the study was mainly exploratory, all of the studied associations were presented, and non-significant paths were also retained in the model.

In the multigroup analysis, the structural invariance of the model was tested (Table 4). According to the results of the nested model comparisons, there were no differences between the groups in structural weights (χ^2^ = 18.25, df = 12, *p* = 0.107). However, structural intercepts (χ^2^ = 26.41, df = 14, *p* = 0.023), means (χ^2^ = 42.22, df = 20, *p* = 0.003), covariances (χ^2^ = 84.48, df = 41, *p* = 0.001) and residuals (χ^2^ = 92.54, df = 44, *p* = 0.001) differed significantly.

**Table 4:** Multiple Group analysis – summary of model comparisons and model fit indices

	χ^2^	df	*p*	NFI Delta-1	χ^2^/df	RMSEA (CI: 90%)	Pclose	NFI	TLI (NNFI)	CFI
Structural weights	18.294	12	0.107	0.024	1.525	0.044 (0.001–0.082)	0.555	0.976	0.944	0.991
Structural intercepts	26.409	14	0.023	0.035	0.023	0.057 (0.09–0.327)	0.327	0.965	0.906	0.982
Structural means	42.219	20	0.003	0.056	0.003	0.064 (0.091–0.181)	0.181	0.944	0.882	0.967
Structural covariances	84.478	41	0.001	0.113	0.001	0.062 (0.081–0.133)	0.133	0.887	0.887	0.936
Structural residuals	92.539	44	0.001	0.123	0.001	0.064 (0.082–0.103)	0.103	0.877	0.883	0.098

Since there was no difference between structural weights in both groups, we present the model of equal structural weights, as the associations in both groups were invariant (Table 5). Higher expected autonomy and expected emotional support from the partner were associated with relationship satisfaction negatively (expected autonomy: Estimate: -0.648, *p* = 0.004; expected emotional support: Estimate: -0.716, *p* = 0.013), while received emotional support (Estimate: 3.067, *p* < 0.001) predicted relationship satisfaction positively. Self-esteem was predicted significantly by expected and received directivity as well as emotional support received from the partner (expected directivity: Estimate 0.34, *p* < 0.001; received directivity: Estimate: -0.355, *p* = 0.02; received emotional support: Estimate: 3.067, *p* = 0.002).

**Table 5:** Explorative path model

Outcomes	Relationship Satisfaction					Self-Esteem				
Predictors (support types)	Estimate	Stand. estimate	S.E	C.R	*p*	Estimate	Stand. estimate	S.E	C.R	*p*
Expected autonomy	-0.648	-0.140^a^; -0.124^b^	0.222	-2.92	0.004	0.41	0.092^a^; 0.095^b^	0.266	1.538	0.124
Received autonomy	0.022	0.004^a^; 0.004^b^	0.257	0.085	0.932	-0.036	-0.007^a^; -0.008^b^	0.305	-0.117	0.907
Expected directive	0.34	0.081^a^; 0.081^b^	0.251	1.356	0.175	-1.158	-0.287^a^; -0.336^b^	0.296	-3.916	< 0.001
Received directive	-0.355	-0.087^a^; -0.086^b^	0.254	-1.401	0.161	0.725	0.184^a^; 0.212^b^	0.301	2.407	0.016
Expected emotional	-0.716	-0.160^a^; -0.129^b^	0.287	-2.495	0.013	0.313	0.073^a^; 0.069^b^	0.337	0.929	0.353
Received emotional	3.067	0.850^a^; 0.770^b^	0.237	12.948	< 0.001	0.854	0.246^a^; 0.261^b^	0.276	3.095	0.002

*C.R*. Critical Ratio

^a^Diagnosed group

^b^Control group

## Discussion

To the best of our knowledge, this is the first SDT-based study examining different types of support from the partner for personal projects of women with and without a BC diagnosis. Moreover, the findings provide new insights about how these support types are associated with relationship satisfaction and self-esteem. Results showed that not only the type of support but the distinction between expected and actually received support should be considered in scientific investigations. The first section of the Discussion examines the differences between the two study groups. Then, as the results showed that associations between personal project support and relationship satisfaction and self-esteem did not differ between the diagnosed and control groups, the pooled results are discussed in the second part of the Discussion.

### Differences between diagnosed and non-diagnosed respondents

Women with BC diagnoses expected and, at the same time, received more autonomy support from their partner than members of the control group. The fact that diagnosed women expect more autonomy support compared to the control group suggests that illness is associated with a greater need to make own decisions about projects. Although having a diagnosis can enhance reassurance seeking or dependence [41] and patients have a higher tendency to disclose self-conscious emotions compared to their partners [18], some studies pointed out that patients with BC endeavor to save their partner from distress caused by the illness [42].

The other difference which can be observed between the two groups is that diagnosed women were less satisfied with their relationship than the control group. The difference may be due to lower levels of physical well-being, such as low libido because of the treatment procedures and less capacity to actively engage in the relationship and accept support from the partner. According to a previous study, patients with BC are more likely to have difficulties in intimate relationships relative to the control group, which can be linked to body image disturbances [7], and they can be characterized by a higher level of avoidant attachment style and lower sexual satisfaction relative to women without a diagnosis [43]. In addition, the associations between experiencing intimacy with the partner and a sense of meaningfulness were significantly weaker in patients with BC compared to women without [7].

Importantly, in the present study, there were no differences between the diagnosed and the control group in the associations of project support types and relationship satisfaction and self-esteem. This association suggests that emotional and directive support for personal projects can contribute equally to the well-being of women with and without BC diagnosis. Thus, these results indicate that, although BC can affect well-being negatively, having the disease itself does not necessarily affect relational functioning. This conclusion contradicts some of the recent findings in the literature (e.g. [44, 45]). However, they are in line with the arguments of Désfalvi and colleagues [43] who conclude that the diagnosis can impact the quality of the relationship but does not necessarily affect its basic functioning. Future research could clarify the conditions under which couples affected by BC are able to maintain the most essential features of their relationship.

### Associations between different types of personal project support as well as relationship satisfaction and self-esteem

Among support types, emotional support was the most consistent predictor of relationship satisfaction and self-esteem. Expected emotional support from the partner correlated with relationship satisfaction negatively, while received emotional support was positively associated with relationship satisfaction. These associations are in line with SDT, suggesting that the partner’s emotional availability and acceptance are essential for relational well-being [28, 19]. At the same time, expectations of higher emotional support may reflect unmet emotional needs in the relationship. Self-esteem was also positively associated with emotional support received from the partner. This result confirms previous studies’ conclusion that the partner’s support can enhance self-esteem and self-acceptance [3]. Furthermore, our results provide novel information, specifically for personal project pursuit, corroborating the importance of the partner’s emotional support.

Beyond emotional support, directivity also proved to be a significant predictor of self-esteem. Higher expected directivity while working on personal projects was associated with lower levels of self-esteem. This association can be interpreted as those with lower of self-esteem levels may need more concrete guidance and advice from their partner and presumably have less trust in their abilities. At the same time, those who received more directivity had higher self-esteem. Similarly, previous studies reached ambiguous conclusions concerning the role of directivity in well-being and functioning. On the one hand, unwanted advice can diminish personal volition [31]. On the other hand, recent investigations showed that directive support could be interpreted as care and attention, and positive control from the partner may enhance well-being and facilitate more favorable health behavior [32]. Women with BC may particularly value directive support as a way of receiving care and attention, because they may experience increased ambivalence and uncertainty.

The positive interpretation of directivity and controversial judgment of autonomy in this sample of Hungarian women complement each other and may reflect culture-specific characteristics. The few studies in Hungarian samples that focused on autonomy versus directive support were conducted in academic settings and showed that not just autonomy support but teachers’ direct control also correlated with self-efficacy [46]. Research also suggests that, contrary to gradual changes in social norms, conformity and low preferences for novelty are still common in Hungarian society as a post-socialist country in transition where autonomy is still an undervalued phenomenon and coincides with a lack of guidance [47]. Neither directivity nor expected and received support had predictive power for relationship satisfaction. According to SDT, control of the environment can have detrimental effects on well-being, and some previous studies found that concrete suggestions can set back well-being and goal internalization [e.g. [5, 29]]. However, in some cases, directive support was not consistently related to relationship satisfaction [30].

Results show a somewhat controversial picture of the partner’s expected and received autonomy support. Relationship satisfaction was negatively predicted by expected autonomy support; thus, women seem to be less satisfied with the relationship if there is a need for getting freedom of choice during personal projects. At the same time, perceived autonomy support did not predict relationship satisfaction and self-esteem. A possible explanation for this can be that the items used for capturing expected and received autonomy were partly interpreted as independence and distance. This explanation fits the findings of Soenens and colleagues [48]; they distinguished between two concepts of autonomy support: the support of personal volition suggested by SDT and the support of independence (the previous proved to be an important predictor of psychosocial adaptive capacity). From this perspective, higher expected autonomy support may gain importance when women want independence and distance from their partners.

### Differences between expected and received support

As expected and received support can be explained in relation to each other, we examined if there were general differences between women’s expected and received support from their partner. Our results support the idea that simultaneously measuring subjective expectations and perceptions of support is a reasonable strategy to collect more nuanced information. Overall, women (diagnosed and control samples collapsed) expected more emotional support but less autonomy support than they received. This association may be interpreted as a particular need for the partner’s relatedness and emotional closeness during working on personal projects [24, 26]. In turn, the lower need for autonomy support from the partner may reflect the interpretation of autonomy as striving for independence [48].

### Limitations

One limitation of our study is that its cross-sectional and correlational nature prevents conclusions being drawn on cause and effect regarding associations between project support and well-being [49]. Another limitation is that only one item was applied for measuring each support type. Although each of these items was rated four times (across the four projects), it may be more beneficial to apply multi-item scales to obtain a more comprehensive picture of the nature and interpretation of support in the future. Moreover, to capture the different types of expected and received support, we developed complex statements specifically for the present study; however, we have not assessed their psychometric properties previously. Therefore, future investigations should confirm the validity of our methods and test the associations we have found using similar assessment techniques. Finally, our study could have included several additional variables to strengthen its explanatory power. For example, future studies may include direct measurement of basic psychological need satisfaction and detailed illness-relevant variables (e.g., having metastasis or not in patients with BC).

## Conclusions

Differentiating between types of partner support is important while accomplishing personal goals during a chronic illness. Among support types, emotional and directive support emerged as the most relevant in our study on women with breast cancer. Results indicate the simultaneous need for autonomous decisions about personal projects and emotional support. In future studies, partners might also be interviewed so that we could better understand both partners’ perspectives [30]. Nevertheless, a general theory of how BC affects communication between couples is yet to be developed and tested [50]. A longitudinal study could explore different stages of the disease, where patients’ experiences may likely go through significant changes [19]. Since the partners’ actual autonomy support for the projects did not predict well-being, future investigations into autonomy support from other sources (e.g., from physicians) could be a promising direction. Concerning potential interventions, our study shows that it would be essential to involve the partner in the adaptation process [5]. For example, discussing personal projects of the couples related to a current life situation, such as a diagnosis of cancer, or other life events, can be used in relationship counseling. Regarding the importance of emotional support, such an intervention for personal project support could help partners to be emotionally supportive. Furthermore, teaching women effective communication skills to express their needs to their partners, such as the need for autonomy or directive guidance, can facilitate a shared understanding of expected and actual support.

## Data Availability

The datasets generated during and/or analyzed during the current study are available from the corresponding author on reasonable request.
